# Brown-Vialetto-Van Laere syndrome

**DOI:** 10.1186/1750-1172-3-9

**Published:** 2008-04-17

**Authors:** Sivakumar Sathasivam

**Affiliations:** 1 The Walton Centre for Neurology and Neurosurgery, Liverpool, UK

## Abstract

The Brown-Vialetto-Van Laere syndrome (BVVL) is a rare neurological disorder characterized by progressive pontobulbar palsy associated with sensorineural deafness. Fifty-eight cases have been reported in just over 100 years. The female to male ratio is approximately 3:1. The age of onset of the initial symptom varies from infancy to the third decade. The syndrome most frequently presents with sensorineural deafness, which is usually progressive and severe. Lower cranial nerve involvement and lower and upper motor neuron limb signs are common neurological features. Other features include respiratory compromise (the most frequent non-neurological finding), limb weakness, slurring of speech, facial weakness, and neck and shoulder weakness. Optic atrophy, retinitis pigmentosa, macular hyperpigmentation, autonomic dysfunction, epilepsy may occur. The etiopathogenesis of the condition remains elusive. Approximately 50% of cases are familial, of which autosomal recessive is suggested. The remaining cases are sporadic. The diagnosis is usually based on the clinical presentation. Investigations (neurophysiological studies, magnetic resonance imaging of the brain, muscle biopsy, cerebrospinal fluid examination) are done to exclude other causes or to confirm the clinical findings. The differential diagnoses include the Fazio-Londe syndrome, amyotrophic lateral sclerosis, Nathalie syndrome, Boltshauser syndrome and Madras motor neuron disease. Treatment with steroids or intravenous immunoglobulin may result in temporary stabilization of the syndrome. However, the mainstays of management are supportive and symptomatic treatment, in particular assisted ventilation and maintenance of nutrition via gastrostomy. The clinical course of BVVL is variable and includes gradual deterioration (almost half of cases), gradual deterioration with stable periods in between (a third of cases) and deterioration with abrupt periods of worsening (just under a fifth of cases). After the initial presentation, one third of patients survive for ten years or longer.

## Definition

The Brown-Vialetto-Van Laere syndrome (BVVL) is a rare neurological disorder of unknown etiology, characterized by progressive pontobulbar palsy associated with sensorineural deafness. It was first described by Brown in 1894 [1], and later by Vialetto and Van Laere in 1936 [2] and 1966 [3] respectively.

## Epidemiology

Fifty-eight cases of BVVL have been reported in just over a century (Additional file 1). Around half of all cases are sporadic [4]. The majority of familial cases demonstrate autosomal recessive inheritance, although autosomal dominant [5,6] or X-linked inheritance [5] has been suggested in a few families. The female to male ratio is approximately 3:1 in reported cases. This may be the result of reporting bias as males tend to be more severely affected and therefore die earlier in life [5-10].

## Clinical description

It is difficult to map out accurately the clinical course of BVVL as most case reports do not give a detailed account of the development of symptoms and signs. However, in the vast majority of cases the first symptom is sensorineural deafness, which is usually progressive and severe. The time between the onset of deafness and the development of other symptoms has been reported to be shorter in males (mean of approximately five years) than in females (mean of almost 11 years) [11]. Very rarely, affected cases do not appear to develop deafness, presumably because these individuals die before the hearing impairment develops [9]. Other initial presenting features include limb weakness [12-15], respiratory compromise [8,9], slurring of speech [16], facial weakness [9], and neck and shoulder weakness [17]. The age of onset of the initial symptom varies from infancy [2] to the third decade [18,19]. In a few cases, an intercurrent event, such as an infection, appears to have precipitated the initial symptom or worsened an existing symptom [2,7,8,12,20,21].

In BVVL, the lower cranial nerves VII to XII are commonly affected, while abnormalities of cranial nerves II to VI occur much less frequently. Cerebellar ataxia was reported in one case [22]. Lower motor neuron (LMN) signs are common in the limbs. Upper motor neuron (UMN) involvement, for example brisk reflexes, clonus and extensor plantar responses, is less frequent [5-11,13,14,19-26]. Sensation is rarely affected, with only one reported case of subjective blunting of pinprick sensation below the knees [16].

Several other neurological features have been seen in patients with BVVL. Abnormalities of the fundi that have been reported include optic atrophy [5,13,20,21,27], retinitis pigmentosa [22] and macular hyperpigmentation [25]. Autonomic dysfunction [2,13,28,29], epilepsy [2,18], mental retardation [1,2,25], reduced horizontal eye movements [6] and tremor [9,25] have also been associated with BVVL.

Of the non-neurological features, respiratory compromise is the most common in BVVL [4-13,15,17,19-21,24,27-33]. Other non-neurological features that have been reported include auditory hallucinations [2], behavioral changes [27], color blindness [20], diabetes insipidus [1], delayed puberty and hypogonadism [16], dysmorphic features [25], gynecomastia [16] and hypertension [11,33]. In some cases, no other associated non-neurological features were reported [34-38].

## Etiopathogenesis

The etiopathogenesis of BVVL remains elusive. Two patients with BVVL have been screened for the mutations associated with common forms of spinal muscular atrophy (SMA), the survival motor neuron gene (*SMA*) and neuronal apoptosis inhibitory protein gene (*NAIP*), but these proved negative (4,39). There are no other published studies on the investigation of the genetic or molecular pathogenesis of BVVL.

## Diagnosis

The diagnosis of BVVL is based on the clinical description of the syndrome, as there is no confirmatory test for the condition. The major features of BVVL are the presence of sensorineural deafness, involvement of lower cranial nerves VII to XII and the presence of LMN, and to a lesser extent UMN, signs in the limbs. However, except for the sensorineural deafness, the presence of the other features can be variable. Respiratory compromise is the most common non-neurological feature in BVVL. The other neurological and non-neurological features seen in patients with BVVL described in the section 'Clinical description' are seen much less frequently.

Investigations are usually done to exclude other causes or confirm the clinical signs of the patients. Neurophysiological studies show changes consistent with chronic [5,7,9,11-14,16,21,22,25,28,33] or active [6-8,15,19,20,24,26,27,29,33] denervation in muscles. Motor nerve conduction velocities are usually normal. Sensory action potentials are rarely reduced [4,6,7]. Visual evoked potentials performed in 15 cases showed normal values in seven patients [9,14,25,28,32,33] and prolonged latencies in the rest [5,7,10,12,13,15,19,21,29]. Brainstem auditory evoked potentials were abnormal when performed in 17 cases [4,5,8,10,12-15,21,25,27,32,33]. When carried out, audiometry universally showed sensorineural deafness. Electroencephalogram may show an excess of theta activity or slow waves [5,20,23,30]. Electrocardiogram showed incomplete right bundle branch block in one case [4]. Polysomnography demonstrated predominantly central sleep apnea with minimal obstructive sleep apnea in one case [32].

Magnetic resonance imaging of the brain may show atrophy of the brainstem [21,25,27] and cerebellum [21,25], or hyperintensity in the brainstem nuclei [10,27], cerebellar peduncles [29], internal capsule [29] or subcortical white matter [29].

Muscle biopsy was carried out on eight patients. In four cases it showed normal muscle histology [6,9,11,27], in one case it showed increased lipid content and myopathic changes (unspecified) [29], while in the remaining cases there was evidence of grouped atrophic fibres suggesting denervation [5,7,12,14,20,22,25,28]. Sural nerve biopsies were undertaken in two patients, with one showing axonal depletion [22], while the other was normal [20].

Cerebrospinal fluid examination in BVVL may show mildly elevated protein content [5,12,13,30,33].

There are few pathological descriptions of BVVL available due to the rarity of the condition [7,12,13,20,30]. There is usually neuronal injury and loss in the III, V, VI and lower cranial nerve nuclei (VII – XII). However, studies in a two-year old boy [7] and a 10-year old girl [30], revealed normal cranial nerve nuclei III, V and VI. Other neuropathological findings include depletion of spinal anterior horn cells [12,13,20,30], degeneration of spinocerebellar [13,20,30] and pyramidal [13,20] tracts, degeneration of cerebellar Purkinje cells [20], and abnormalities in the substantia nigra [12,13], locus coeruleus [12,20], olives [12,13], cuneate nucleus [12], gracile nucleus [12,20], ambiguous nucleus [7,12,20], dorsal nucleus of Clarke [12,20,30], fastigial nucleus [20], lateral lemnisci [12,20], medial longitudinal fasciculus [12], trapezoid body [12,13], optic pathways [12] and solitary tract [12,20].

## Differential diagnosis

**Figure 1:**
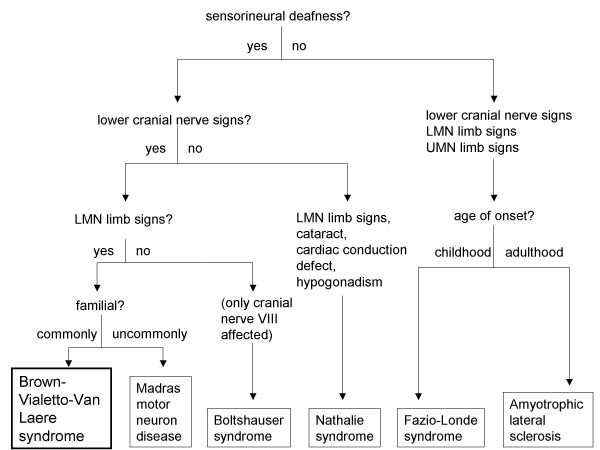
Diagnostic algorithm for BVVL.

There are several conditions that closely resemble BVVL and that should always be considered in the differential diagnosis. It would be unrealistic to expect to make a diagnosis of BVVL in a patient who initially presents with just sensorineural deafness. The development of other cranial nerve and limb involvement in conjunction with sensorineural deafness is likely to be needed to secure the diagnosis with any degree of confidence. Figure 1 provides a diagnostic algorithm to aid in the diagnosis of this rare disorder.

Perhaps the most closely related condition is the progressive bulbar paralysis of Fazio-Londe [40], where the only distinguishing feature from BVVL is the absence of deafness. In fact, in the case reported by Voudris and colleagues [8], deafness was not clinically recognized and was only picked up by brainstem auditory evoked potential testing. In addition, in two of the four cases by Dipti and colleagues [9] who presented early in life, deafness was not thought to be present, possibly implying that the patients died before the deafness had developed.

Amyotrophic lateral sclerosis (ALS) is another alternative diagnosis. However, it does not usually present at a young age and sensorineural deafness is not a feature of this condition.

Another differential of BVVL is the Nathalie syndrome, which is a rare condition characterized by deafness in conjunction with spinal muscular atrophy, cataract, cardiac conduction defects and hypogonadism [41]. Interestingly, one reported case of BVVL had a partial right bundle branch block on electrocardiography [4] and another had hypogonadism [16].

The Boltshauser syndrome, which is characterized by distal muscular atrophy with vocal cord paralysis and sensorineural hearing loss, is also very similar to BVVL [42]. However, in the former, the brainstem signs are restricted to vocal cord paralysis and the inheritance is likely to be autosomal dominant. Autosomal dominant inheritance is very uncommon in BVVL, with only two possible families reported [5,6].

The Madras motor neuron disease (MMND) is another condition closely related to BVVL [43]. MMND is characterized by wasting and weakness of limb muscles, sensorineural deafness and multiple cranial nerve palsies usually affecting cranial nerves VII, IX and XII. Dysfunction of cranial nerves III and VI has not been reported in MMND [44]. Only about 15% of cases of MMND are familial [44], compared to 50% in BVVL [4].

## Management and treatment

There is no specific treatment for BVVL. Steroids and immunoglobulins have been tried in several cases. Temporary stabilization was reported two patients. In the first, steroids stabilized the condition temporarily for at least eight months [7]. Stabilization of the condition for a year was seen in another patient who received intravenous immunoglobulin [11]. However, two other patients who received intravenous immunoglobulin failed to show any improvement [28,32].

Supportive care and symptomatic treatment are the mainstays of management for BVVL. In a rare condition like BVVL, evidence for the effectiveness of these measures is anecdotal as it would be impossible to carry out randomized controlled trials with the small numbers of patients available. However, the benefit from experience of using these methods in other similar conditions, in particular ALS, can be extrapolated for BVVL: for example, in ALS assisted ventilation and maintenance of nutrition have been proven to improve survival [45].

In BVVL, assisted ventilation has been shown to be useful when respiratory compromise is a major feature. Long-term nocturnal ventilatory support was beneficial in some cases [5,6,9,15,19]. Tracheostomy and full ventilatory support was helpful in other cases [8-10,20,24,28,29,31,32]. In patients where dysphagia is a major problem, gastrostomy can alleviate its symptoms and maintain good nutritional status [6,8-10,13,28].

## Prognosis

The clinical course of BVVL is variable in the 58 cases reported in the literature (Additional file 1). In 26 cases (45%), patients had gradual deterioration (GD), while in 19 cases (33%), there was gradual deterioration with stable periods in between (GDS). Ten patients (17%) had deterioration with abrupt periods of worsening (DW). The clinical course was not known in three cases (5%). At least 21 patients (36%) survived for 10 or more years after the initial symptom. Twenty three patients (40%) survived for five or less years after the onset of the first symptom.

## Unresolved questions

The key unanswered question in BVVL is the molecular pathogenesis of the condition. Although the syndrome is very rare, advances in molecular biotechnology offer hope that the underlying pathogenesis of the disease will be unravelled in the near future.

It is likely that we still do not know the full clinical spectrum of BVVL. Thus, it is important that as more and more cases of BVVL are recognized, these cases continue to be reported in the literature to enhance our knowledge and understanding of this rare progressive neurological disorder. This will enable affected individuals and families to be provided with a better idea of the clinical course, prognosis and management of this debilitating condition.

## Competing interests

The author(s) declare that they have no competing interests.

## Authors' contributions

SS identified all the titles and abstracts for the review and wrote the review.

## Supplementary Material

Additional file 1 Published cases of Brown-Vialetto-Van Laere syndrome (BVVL). The data provided represent the gender, clinical features, diseases course and duration of BVVL in 58 published cases. Click here for file
