# Supplementary material for: Brown-Vialetto-Van Laere syndrome
Source: Orphanet J Rare Dis. 2008 Apr 17;3:9. doi: 10.1186/1750-1172-3-9 (PMC2346457; doi:10.1186/1750-1172-3-9)
Supplement: Additional file 1 — Published cases of Brown-Vialetto-Van Laere syndrome (BVVL). The data provided represent the gender, clinical features, diseases course and duration of BVVL in 58 published cases. [file 1750-1172-3-9-S1.doc]

| **Published cases of Brown-Vialetto-Van Laere syndrome** | | | | | | |
| --- | --- | --- | --- | --- | --- | --- |
| **Reports** | **Gender** | **Initial symptoms (age, years)** | **Cranial nerves involved** | **Associated clinical features** | **Disease course** | **Disease duration;**  **survival status (years)** |
| Brown 1894 [1] | M | Deafness (12) | VII – XII | Mental retardation, diabetes insipidus | GDS | 3 y; alive at age 15 |
| Vialetto 1936 [2] | F | Deafness (16) | III, V, VII – XII | Autonomic dysfunction, mental retardation, epilepsy | GD | 25y; alive at age 41 |
|  | F | Deafness (infancy) | VII – XII | Auditory hallucinations | GDS | 34y; alive at age 34 |
|  | F | Deafness (age unknown) | VII – XII |  | GDS | unknown; alive at age 35 |
| Van Laere 1966 [3] | F | Deafness (10) | VII – XII |  | GD | 1y; alive at age 11 |
|  | F | Deafness (9) | VII – XII |  | GDS | 13y; alive at age 22 |
|  | F | Deafness (11) | VII, VIII |  | GDS | 7y; alive at age 18 |
|  | F | Deafness (12) | VII, VIII |  | GDS | 4y; alive at age 16 |
| Van Laere 1967 [34] | M | Deafness (13) | VI – XII |  | DW | 14y; alive at age 27 |
| Arnould 1968 [35] | F | Deafness (7) | VI – XII |  | DW | 29y; alive at age 36 |
| Trillet 1970 [36] | M | Deafness (19) | VII – XII |  | DW | 5y; alive at age 24 |
| Boudin 1971 [37] | F | Deafness (11) | V, VII – XII |  | GDS | 11y; alive at age 22 |
|  | F | Deafness (14) | VIII – XII |  | GDS | 10y; alive at age 24 |
| Serratrice 1972 [38] | F | Deafness (15) | III, VII – XII |  | GD | 6y; alive at age 21 |
| Van Laere 1977 [18] | F | Deafness (31) | VII – XII | Epilepsy | GDS | 16y; alive at age 47 |
| Alberca 1980 [22] | F | Deafness (5) | III, V, VII – XII | Cerebellar ataxia, retinitis pigmentosa, UMN signs | GD | 22y; died at age 27 |
| Gallai 1981 [7] | F | Deafness (2) | VII – XII | Respiratory compromise, UMN signs | DW | 13y; alive at age 15 |
|  | M | Deafness (1) | V, VII – XII |  | DW | 1y; died at age 2 |
|  | F | Deafness (6) | VII – XII | Respiratory compromise, UMN signs | DW | 7y; alive at age 13 |
| Brucher 1981 [20] | F | Deafness (17) | II, III, V – XII | UMN signs | GD | 8y; died at age 25 |
|  | F | Deafness (12) | II, V, VII – XII | Respiratory compromise, UMN signs | GD | 0y; died at age 12 |
| Rosemberg 1982 [30] | F | Deafness (9) | VII – XII | Respiratory compromise | GD | 1y; died at age 10 |
| Tavares 1985 [23] | F | Deafness (9) | VII, VIII, XI, XII | UMN signs | GD | 10y; alive at age 19 |
| Summers 1987 [12] | F | Limb weakness (12) | VII – X | Respiratory compromise | GDS | 2y; alive at age 14 |
| Hawkins 1990 [5] | F | Deafness (12) | II, VII – XII | Respiratory compromise, UMN signs | GD | 5y; died at age 17 |
| Abarbanel 1991 [13] | F | Limb weakness (12) | II, III, VII,  VIII, X – XII | Autonomic dysfunction, respiratory compromise, UMN signs | GD | 12y; alive at age 24 |
| Piccolo 1992 [19] | M | Deafness (30) | II, V – X, XII | Respiratory compromise, UMN signs | DW | 8y; alive at age 38 |
| Francis 1993 [21] | M | Deafness (8) | II, V – XII | Color blindness, respiratory compromise, UMN signs | DW | 10y; died at age 18 |
| Davenport 1994 [28] | F | Deafness (age unknown) | VIII – X, XII | Autonomic dysfunction, respiratory compromise | GD | unknown; alive age 18 |
| De Oliveira 1995 [33] | F | Deafness (7) | VII – XII |  | unknown | 5y; alive at age 12 |
|  | M | Deafness (15) | VII, VIII | Hypertension | unknown | 2y, alive at age 17 |
| Puri 1996 [24] | M | Deafness (4) | V, VII – X, XII | Respiratory compromise, UMN signs | GD | 7y; alive at age 11 |
| Sztajzel 1998 [11] | F | Deafness (10) | VII, VIII, XI, XII | Hypertension, respiratory compromise, UMN signs | GDS | 32y; died at age 42 |
| Mégarbané 2000 [4] | M | Deafness (2) | VII, IX – XII |  | GD | 9y; died at age 11 |
|  | M | Deafness (unknown) | VII – XII | Respiratory compromise | DW | unknown; died at age 7 |
|  | M | Deafness (3) | VII – XII |  | GD | 3y; alive at age 6 |
| Sathasivam 2000 [16] | M | Slurring of speech (20) | VII – X, XII | Delayed puberty, hypogonadism, gynecomastia | GD | 2y; alive at age 22 |
| Voudris 2002 [8] | M | Respiratory compromise (1) | II – IV, VI – XII | UMN signs | GD | 9m; died at age 1 |
| Introini 2003 [29] | M | Deafness (24) | VII – XII | Autonomic dysfunction, respiratory compromise | DW | 12y; alive at age 36 |
| Koc 2003 [25] | M | Deafness (7) | VII, VIII, IX, X, XII | Dysmorphic features, mental retardation, UMN signs, tremor, macular hyperpigmentation | GD | 10y; alive at age 17 |
| Aydin 2004 [14] | F | Limb weakness (9) | VII – XII | UMN signs | GDS | 5y; alive at age 14 |
| RamachandranNair 2004 [26] | F | Deafness (7) | VIII – XII | UMN signs | GD | 8y; alive at age 15 |
| De Grandis 2005 [6] | F | Deafness (age unknown) | III, VII – XII | Respiratory compromise, reduced horizontal eye movements, UMN signs | GDS | unknown; alive at age 48 |
| Dipti 2005 [9] | M | Facial weakness, respiratory compromise (1) | VII, IX, X |  | GD | 6m; died at age 1 |
|  | F | Respiratory compromise (1) | VII, IX – XII |  | GD | 7m; died at age 1 |
|  | F | Respiratory compromise (5) | III – VIII | UMN signs, tremor | GDS | 9y; alive at age 14 |
|  | F | Deafness, respiratory compromise (7) | VII – XII | UMN signs | GD | 3y; died at age 10 |
| Nemoto 2005 [15] | F | Deafness, limb weakness (15) | III, VII, X – XII | Respiratory compromise | GD | 45y; alive at age 60 |
| Prabhu 2005 [31] | F | Deafness (28) | VII, VIII, X | Respiratory compromise | GDS | 7y; alive at age 38 |
| Descatha 2006 [17] | F | Deafness, neck & shoulder weakness (12) | VII – XI | Respiratory compromise | GDS | 10y; died at age 22 |
| Koul 2006 [10] | M | Deafness (9) | V, VII – XII | UMN signs | GD | 1y; died at age 10 |
|  | F | Deafness (9) | VII – XII | Respiratory compromise, UMN signs | GD | 2y; died at age 11 |
|  | F | Deafness (11) | V, VII – XII | UMN signs | unknown | 5y; alive at age 16 |
| Malheiros 2007 [27] | F | Deafness (20) | II, VIII – XII | Behavioral changes, respiratory compromise | GDS | 35y; alive at age 55 |
|  | F | Deafness (18) | VII – XII |  | GDS | 35y; alive at age 53 |
|  | F | Deafness (12) | VIII – XII | Behavioral changes, respiratory compromise | GDS | 36y; alive at age 48 |
|  | F | Deafness (18) | VIII |  | GD | 5y; alive at age 23 |
| Miao 2007 [32] | F | Deafness (14) | VII, IX, X, XII | Respiratory compromise | GD | 2y; died at age 16 |
